# DNA-Engineered Coating for Protecting the Catalytic Activity of Platinum Nanozymes in Biological Systems

**DOI:** 10.3390/bios15040205

**Published:** 2025-03-21

**Authors:** Lei Ren, Xia Liu, Shuai Tang, Yue Wang, Miao Yang, Linjie Guo, Jiang Li, Kai Jiao, Lihua Wang

**Affiliations:** 1 Division of Physical Biology, CAS Key Laboratory of Interfacial Physics and Technology, Shanghai Institute of Applied Physics, Chinese Academy of Sciences, Shanghai 201800, China; renlei@sinap.ac.cn (L.R.); wangyue0107@sinap.ac.cn (Y.W.); 2 University of Chinese Academy of Sciences, Beijing 100049, China; 3 Institute of Materiobiology, College of Sciences, Shanghai University, Shanghai 200444, China; xliusinap@163.com (X.L.); stang@shu.edu.cn (S.T.); 17837702067@shu.edu.cn (M.Y.); guolinjie@shu.edu.cn (L.G.); lijiang80@shu.edu.cn (J.L.); 4 Xiangfu Laboratory, Jiaxing 314102, China; 5 Shanghai Collaborative Innovation Center of Intelligent Sensing Chip Technology, Shanghai University, Shanghai 200444, China

**Keywords:** nanozyme, DNA coating, platinum nanoparticles, miRNA detection

## Abstract

Nanozymes, exemplified by metal nanoparticles, have shown promise in the fields of biological diagnostics and therapeutics. However, their practical application is often hindered by aggregation or deactivation in complex biological systems. Here, we develop a DNA-engineered nanozyme coating to preserve the peroxidase-like catalytic activity of platinum nanoparticles in complex biological environments. We employed thiol-modified single-stranded DNA to coat the platinum nanoparticles through metal–sulfur interaction. We found that the negatively charged DNA coating prevents the aggregation of platinum nanoparticles in high-salt environments. Moreover, the DNA coating functions as a molecular sieve, inhibiting non-specific protein adsorption while preserving substrate access to the catalytic interface, thus sustaining high peroxidase-like catalytic activity in serum. As a proof of concept, we demonstrate miRNA detection in serum samples with a detection limit of 1 fM. This approach offers a versatile strategy for molecular diagnostics of nanozymes in complex biological environments.

## 1. Introduction

Nanozymes are synthetic nanomaterials that mimic the catalytic functions of natural enzymes [1,2,3]. Representative examples include platinum, gold, silver, palladium, and iron oxide nanoparticles, exhibiting peroxidase [4], catalase [5], and superoxide dismutase-like activities [6]. Compared to protein-based natural enzymes, nanozymes demonstrate superior structural tunability, enhanced stability, and lower production costs. These advantages have enabled their widespread applications in biological diagnostics and therapeutics [7,8], environmental remediation [9,10] and agricultural sciences [11]. However, the catalytic performance of nanozymes is susceptible to environmental interference. For instance, the nonspecific adsorption of biomolecules in complex biological systems can block active sites and compromise enzymatic activity [12]. Surface engineering represents a pivotal strategy for modulating nanozyme performance by precisely regulating surface physicochemical properties, active site accessibility, and anti-fouling capacity, thereby enhancing catalytic efficiency, selectivity, and operational stability [13]. Conventional coating materials including chitosan [14], polyethylene glycol [15], mesoporous silica, and metal–organic frameworks [16] primarily provide physical protection and improve colloidal stability. Nevertheless, these materials suffer from critical limitations: lack of precise control over coating thickness and density leading to active site occlusion, insufficient biocompatibility, and absence of inherent molecular recognition capabilities requiring additional functionalization steps.

DNA-based surface engineering offers distinct advantages through the creation of spherical nucleic acids (SNAs)—nanoparticles densely functionalized with oligonucleotides [17]. The DNA shell enables programmable control over coating density and thickness [18,19], while conferring molecular recognition capabilities and anti-fouling properties through steric and electrostatic effects. This unique architecture prevents cation-mediated aggregation and nonspecific protein adsorption. SNAs have demonstrated remarkable potential in biosensing [20], gene regulation [21,22] and bioimaging [23].

**Figure 1:**
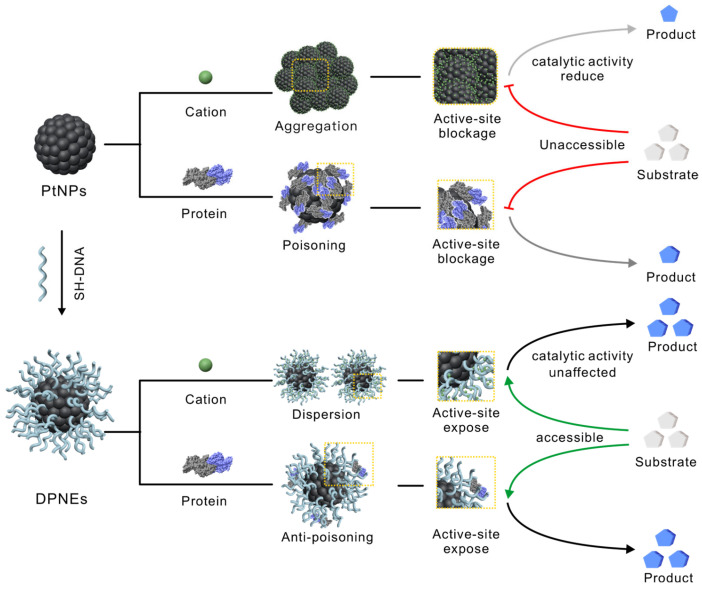
DNA-engineered coatings for the protection of platinum nanozyme catalytic activity in biological systems. The DNA coating on the surface of DPNEs can bind cations in high-salt solutions, maintaining the monodispersity of nanoparticles while also acting as a molecular sieve. This suppresses protein adsorption onto DPNEs, ensuring substrate accessibility to the catalytic interface and preserving their catalytic activity in complex biological environments.

Herein, we demonstrate that DNA shells can serve as engineered surface coatings for nanozymes. Our DNA-coated platinum nanozymes (DPNEs) exhibit exceptional colloidal stability under high ionic strength conditions and effectively resist protein fouling. The densely packed DNA layer functions as a molecular sieve, selectively excluding macromolecules while maintaining accessibility to small substrate molecules (Figure 1). This engineered interface preserves peroxidase-like activity in serum-containing environments. Leveraging these properties, we achieved ultrasensitive detection of HFpEF-associated miRNAs in serum with a detection limit of 1 fM.

## 2. Materials and Methods

### 2.1. Materials and Reagents

In this study, the key experimental materials were sourced as follows: DNA oligonu-cleotide chains and microRNA sequences, phosphate-buffered saline (PBS) and 5× TBE buffer (Shenggong Biotech Co., Ltd., Shanghai, China). Streptavidin magnetic beads (concentration of 10 mg/mL, diameter 1 μm, Nanodongfang Biotechnology Co., Ltd., Nanjing, China); reagents such as 3,3′,5,5′-tetramethylbenzidine (TMB), tris (hydroxyme-thyl) aminomethane (Tris), sodium chloride (NaCl), hydrochloric acid (HCl),magnesium chloride (MgCl_2_), ethylenediaminetetraacetic acid (EDTA), dimethyl sulfoxide (DMSO), hydrogen peroxide (H_2_O_2_), acetate buffer (pH = 4.5), bovine serum albumin (BSA), and serum (Sinopharm Chemical Reagent Co., Ltd., Shanghai, China); and platinum nanoparticles (concentration 0.05 mg/mL, diameter 30 nm, Sigma-Aldrich, St. Louis and Burlington, MA, USA). Additionally, the composition of the buffers used in the experiments is clearly defined; the binding buffer contains 5 mM Tris-HCl, 0.5 mM EDTA and 1 M NaCl, with the pH adjusted to 7.4, and the reaction buffer contains 50 mM Tris-HCl, 140 mM NaCl and 1 mM MgCl_2_, with a pH of 7.4. All chemicals were used without additional purification, and all solutions were prepared using the Millipore system (Darmstadt, Germany).

### 2.2. Synthesis of DNA-Coated Platinum Nanozymes (DPNEs)

The specific procedure for synthesizing DNA-functionalized platinum nanozymes (DPNEs) is as follows: Accurately measure 900 μL of PtNPs at a concentration of 50 μg/mL, 2 μL of PolyT-SH at a concentration of 100 μM and 100 μL of 5× TBE buffer. After thoroughly mixing the three components, vortex the mixture to ensure homogeneity. Then, freeze the mixture at −80 °C for 4 h. After thawing to room temperature, immediately centrifuge the mixture at 4 °C and 7000 rpm for 15 min. After centrifugation, discard the supernatant and wash the precipitate three times with 0.5× TBE buffer to remove any unbound DNA. Finally, resuspend the precipitate in 200 μL of 0.5× TBE solution and store it properly for use in subsequent experiments. The concentration of PtNPs/DPNEs was determined by measuring the absorbance at 350 nm using UV–visible spectroscopy (UV-3010 UV–visible spectrometer, Hitachi, Tokyo, Japan); the zeta potential and particle size were characterized by dynamic light scattering (BeNano 180 Zeta Pro, Bettersize Instruments Co., Ltd., Dandong, China).

### 2.3. Transmission Electron Microscopy (TEM) Characterization

Deposit 10.0 μL of a 50 μg/mL solution of the PtNPs/DPNEs samples onto the carbon film surface and leave it to adsorb to the surface for 15 min. Use a filter paper to wick the sample drop and then rinse the carbon film surface with 20 μL of Milli-Q water to remove excess salts. Deposit 10 μL of 1% (wt/vol) aqueous uranyl acetate solution onto the carbon film surface. Stain the samples for 2 min. Remove most of the uranyl acetate solution with a filter paper, while keeping a thin layer of solution on the surface, and allow the copper grids to dry at RT. Then, image the samples with TEM (Tecnai TF20 microscope, FEI, Hillsboro, OR, USA) operated at an acceleration voltage of 200 kV.

### 2.4. Metal Salt Ion, BSA and Serum Treatment of PtNPs/DPNEs

For the sodium ion treatment of PtNPs/DPNEs, accurately measure 300 μL of PtNPs/DPNEs at a concentration of 50 μg/mL, 100 μL of 5 M NaCl and 100 μL of 0.5× TBE. After thoroughly mixing the components, incubate the mixture at room temperature while shaking at 400 rpm for 6 h. During this process, the concentration of sodium ions can be effectively controlled by precisely adjusting the NaCl solution concentration. For the magnesium ion treatment of PtNPs/DPNEs, the procedure is similar to that for sodium ion treatment.

For the BSA treatment of PtNPs/DPNEs, accurately measure 300 μL of PtNPs/DPNEs at a concentration of 50 μg/mL, 80 μL of BSA at a concentration of 5 mg/mL and 120 μL of PBS, and then mix the components. For the serum treatment of PtNPs/DPNEs, measure 300 μL of PtNPs/DPNEs at a concentration of 50 μg/mL, 80 μL of serum and 120 μL of PBS, and mix. Both mixtures under these treatment conditions should be incubated at 37 °C for 1 h, and then transferred to 4 °C and centrifuged at 7000 rpm for 15 min. After centrifugation, discard the supernatant and wash the precipitate twice to thoroughly remove any unbound proteins. Finally, resuspend the precipitate in 500 μL of PBS for use in subsequent experimental steps.

### 2.5. Peroxidase Activity Measurement Method

This study employed a colorimetric method to measure the peroxidase activity of PtNPs. TMB was used as the chromogenic substrate, and during the reaction process, hydroxyl radicals (OH) generated in situ promote the conversion of TMB to oxidized TMB (oxTMB), which exhibits a characteristic absorption peak at 652 nm. The specific measurement steps are as follows: Accurately add 10 μL of 10 mM TMB, 10 μL of 30 μg/mL PtNPs/DPNEs and 10 μL of 1 M H_2_O_2_ sequentially into 170 μL of sodium acetate buffer (pH = 4.5). Allow the reaction to proceed for 1 to 5 min. During this process, the absorbance is directly proportional to the amount of oxTMB produced. Using the same method, the catalytic activity of PtNPs treated with different salt concentrations or proteins can be measured. It is important to note that all operations were performed in a 96-well plate and detected using an Microplate reader (Synergy H1, BioTek, Winooski, VT, USA).

In addition, by adding TMB solutions of different concentrations, the kinetic properties of PtNPs/DPNEs and PtNPs /DPNEs treated with various salt concentrations or proteins can be evaluated. Based on the Michaelis–Menten equation and the kinetic curves, the Michaelis–Menten constant can be calculated. Error bars are mean ± s.d. (*n* = 3).

### 2.6. Methods and Procedures for miRNAs Detection

First, measure 10 μL of streptavidin-modified magnetic nanoparticles (concentration: 10 mg/mL) and dilute them with the binding buffer, followed by five rounds of washing to thoroughly remove the stabilizer. Next, add 50 μL of a 1 μM biotin-capture DNA solution (dissolved in binding buffer) to the processed magnetic nanoparticles (MNPs), and incubate at 37 °C for 1 h. After completing the magnetic separation, wash the biotin-capture DNA-modified magnetic beads five more times, and finally resuspend them in 20 μL of reaction buffer for use in subsequent steps.

Mix 10 μL of biotin-capture DNA-modified MNPs (concentration 10 mg/mL), 20 μL of DPNEs (concentration 150 μg/mL) and 60 μL of reaction buffer thoroughly. Then, add microRNA diluted to different concentrations in 10 μL of serum. Incubate the mixture at 27 °C, shaking at 1500 rpm for 2 h. Afterward, wash the mixture 5–7 times with the reaction buffer, and finally resuspend it in 10 μL of reaction buffer.

Last, 10 μL of 10 mM TMB and 10 μL of prepared MNPs were added to 70 μL of acetate buffer (pH = 4.5) in sequence, and finally 10 μL of 1 M H_2_O_2_ was added. The reaction was carried out for 10 min. The whole process was strictly carried out in a 96-well plate, and the detection was also carried out by enzyme-linked immunosorbent assay.

## 3. Results and Discussion

### 3.1. Design, Synthesis and Structural Characterization of DNA Coating on PtNPs

PtNPs have been extensively utilized in molecular diagnostics due to their exceptional peroxidase-like catalytic properties [24,25]. We constructed DPNEs through a well-established SNA synthesis protocol [26]. Briefly, PtNPs were incubated with thiol-terminated oligonucleotides (sequences detailed in Table S1), followed by rapid freeze–thaw cycles to achieve high-density DNA surface conjugation, forming DNA-encapsulated nanozymes.

**Figure 2:**
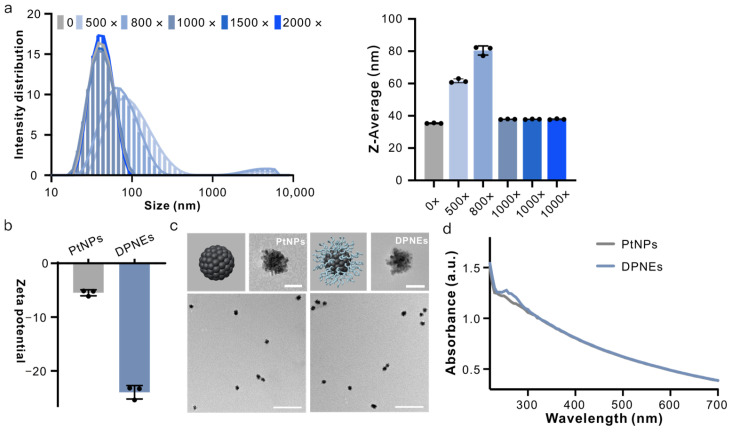
Design, synthesis and characterization of DPNEs. (**a**) Hydrodynamic radius distribution and statistical chart of PtNPs and DPNEs assembled with varying DNA ratios; (**b**) surface potential of PtNPs and DPNEs; (**c**) TEM images of PtNPs and DPNEs; (**d**) UV–visible absorption spectra of PtNPs and DPNEs. Error bars, mean ± s.d. (*n* = 3). Scale bar, 20 nm, 200 nm.

Initial synthesis optimization involved varying DNA/PtNP molar ratios (500:1, 800:1, 1000:1, 1500:1 and 2000:1, termed 500×, 800×, 1000×, 1500× and 2000×). Dynamic light scattering (DLS) revealed distinct hydrodynamic diameter distributions: 500× and 800× DPNEs exhibited size increases to 80.47 ± 2.82 nm and 61.65 ± 1.22 nm, respectively, compared to pristine PtNPs (35.40 ± 0.17 nm), with a bimodal distribution above 1000 nm. While molar ratios exceeded 1000×, DPNEs maintained monodispersity (~37.88 ± 0.18 nm), matching PtNPs’ dimensions (Figure 2a). Subsequent characterization confirmed successful DNA coating; the zeta potential shifted from −5.47 ± 0.55 mV (PtNPs) to −23.97 ± 1.24 mV (DPNEs) (Figure 2b), TEM imaging revealed a low-contrast corona surrounding Pt cores (Figure 2c) and UV–vis spectroscopy showed characteristic DNA absorption at 260 nm (Figure 2d). Following confirmation of DNA coating formation that preserves PtNP monodispersity, we systematically investigated how coating density and thickness modulate peroxidase-mimetic activity. A comparative analysis of DPNEs fabricated with varying DNA/PtNP molar ratios revealed an initial activity reduction upon DNA coating, followed by complete restoration to bare PtNP-level performance at ratios >1000:1, with activity stabilization persisting across higher ratios (Figure S1). The parallel evaluation of DNA length effects (T6, T14, T18, T24) demonstrated equivalent catalytic efficiencies regardless of oligonucleotide length (Figure S1). This empirical optimization identified T14 sequences at 1000:1 molar ratio as the optimal configuration, achieving maximal colloidal stability without compromising catalytic competence while maintaining cost-effectiveness through minimized DNA consumption.

### 3.2. DNA-Coated PtNPs Can Resist High Salt Concentrations

**Figure 3:**
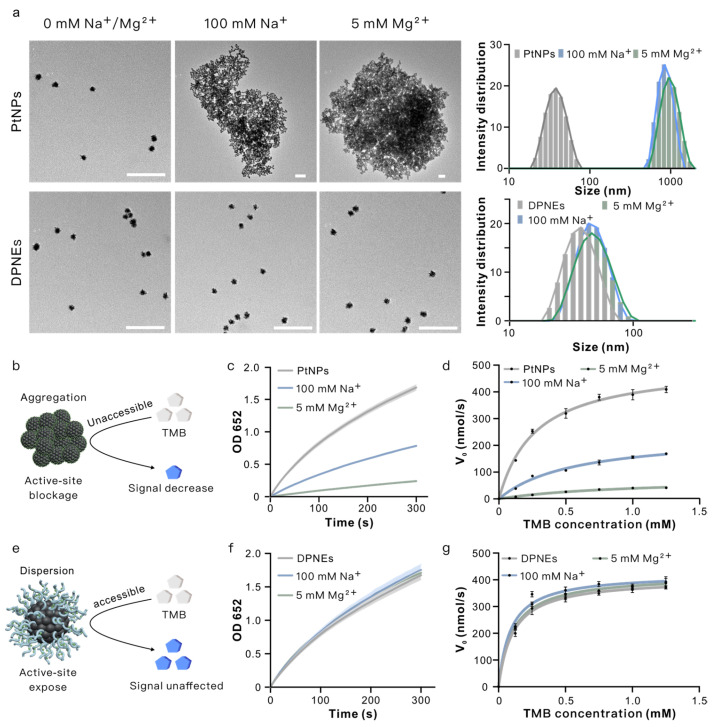
DPNEs resist catalytic activity loss induced by aggregation in high-salt environments. (**a**) TEM images and hydrodynamic radius distribution of PtNPs and DPNEs in the presence and absence of cationic solutions; (**b**) schematic of catalytic activity decline due to aggregation of PtNPs in high-salt environments; (**c**) catalytic reaction kinetics of PtNPs; (**d**) Michaelis–Menten fitting curve for PtNPs; (**e**) schematic of DPNEs resisting aggregation in high-salt environments to maintain catalytic activity; (**f**) catalytic reaction kinetics of DPNEs; (**g**) Michaelis–Menten fitting curve for DPNEs. Error bars, mean ± s.d. (*n* = 3). Scale bar, 200 nm.

We then investigated the salt tolerance of DPNEs using physiologically relevant ion concentrations (100 mM Na^+^, 5 mM Mg^2+^). TEM and DLS analyses demonstrated that PtNPs aggregated severely in high-salt environments (hydrodynamic radius increasing from 35.4 nm to 965.5 nm and 1102.33 nm), while DPNEs maintained colloidal stability (from 37.79 nn to 43.02 nm and 41.63 nm) even at extreme ionic strengths (1 M Na^+^, 40 mM Mg^2+^) (Figure 3a and Figure S2, Table S5). Catalytic performance evaluation through TMB oxidation kinetics revealed critical differences: PtNPs exhibited salt-dependent activity loss with Km values increasing from 0.248 ± 0.026 mM (deionized water) to 0.565 ± 0.106 mM (100 mM Na^+^), 0.975 ± 0.191 mM (5 mM Mg^2+^) and Vmax decreasing from 489.435 ± 13.251 nM/s to 242.462 ± 19.528 nM/s 77.470 ± 8.192 nM/s (Figure 3c,d, Table S2). In contrast, DPNEs maintained consistent kinetic parameters across conditions (Km: 0.108 ± 0.017, 0.090 ± 0.021, 0.107 ± 0.005 mM; Vmax: 404.923 ± 12.958, 423.382 ± 18.002, 417.231 ± 3.692 nM/s) (Figure 3e,f and Figure S3, Table S3).

We propose that in high-salt solutions, the negatively charged PtNPs undergo cation-mediated aggregation, which blocks access of the substrate TMB to catalytic active sites on the nanozyme surface, thereby reducing catalytic activity (Figure 3b). In contrast, the DPNE surface densely coated with DNA exhibits enhanced negative surface potential. Cations in the saline environment preferentially interact with the DNA shell rather than inducing particle aggregation, enabling the DPNEs to maintain a monodisperse state with fully exposed active sites (Figure 3e). This structural preservation ensures sustained high catalytic efficiency under physiologically relevant ionic conditions. We also used palladium nanoparticles (PdNPs) for validation. The DNA coating preserved catalytic performance across all tested conditions (Figure S4), demonstrating the broader applicability of DNA-mediated nanozyme engineering.

### 3.3. DNA Coating Serves as a Molecular Sieve in Serum

**Figure 4:**
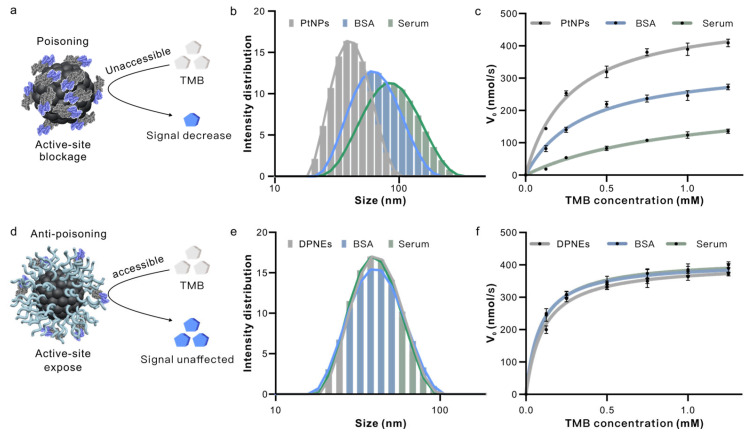
DPNEs resist catalyst poisoning and maintain catalytic activity. (**a**) Schematic of catalytic activity loss of PtNPs due to protein adsorption in serum; (**b**) hydrodynamic radius distribution of PtNPs in serum; (**c**) Michaelis–Menten fitting curve for PtNPs; (**d**) schematic of DPNEs resisting protein adsorption in serum to maintain catalytic activity; (**e**) hydrodynamic radius distribution of DPNEs in serum; (**f**) Michaelis–Menten fitting curve for DPNEs. Error bars, mean ± s.d. (*n* = 3).

We next investigated the DPNEs’ resistance to catalytic deactivation caused by nonspecific biomolecular adsorption in complex biological environments. Using bovine BSA as a model protein and serum as a realistic biological matrix, we observed distinct performance differences between PtNPs and DPNEs. DLS revealed substantial increases in hydrodynamic radius for PtNPs when exposed to BSA (55.767 nm) and serum (71.457 nm) compared to deionized water (35.4 nm), while DPNEs maintained consistent dimensions (37.79, 37.91, 37.28 nm) across all conditions (Figure 4b,e), demonstrating effective anti-fouling capability.

Catalytic kinetics analysis showed progressive deterioration of PtNP performance in protein-containing environments; Km values increased from 0.248 ± 0.026 mM (DI water) to 0.358 ± 0.053 mM (BSA) and 0.987 ± 0.192 mM (serum), with corresponding Vmax decreases from 489.435 ± 13.251 nM/s to 348.718 ± 18.010 nM/s and 245.333 ± 25.838 nM/s (Figure 4c, Table S4). In contrast, DPNEs maintained stable kinetic parameters (Km: 0.108 ± 0.017, 0.093 ± 0.009, 0.0857 ± 0.008 mM; Vmax: 404.923 ± 12.958, 418.872 ± 7.590, 409.436 ± 6.523 nM/s) regardless of environmental complexity (Figure 4f, Table S4). This stability originates from the DNA shell’s molecular sieving effect, which selectively excludes macromolecular interferents while permitting substrate access to catalytic sites (Figure 4a,d).

### 3.4. Detection of miRNAs in Serum Samples

As a proof of concept, we demonstrated the detection of miRNA in serum samples using our DPNEs. Heart failure (HF) is a global health issue, characterized by high incidence and mortality rates, and places a significant burden on healthcare systems. The current clinical diagnosis of HF relies on a combination of cardiac imaging and plasma natriuretic peptide levels. However, the diagnosis of heart failure with preserved ejection fraction (HFpEF), a compensatory form of heart failure, remains challenging due to the limited sensitivity of both cardiac imaging and physiological tests [27,28]. The concentration of miRNAs in blood is strongly correlated with the progression of both acute and chronic diseases, and miRNAs exhibit superior stability in stored blood samples. These characteristics make miRNAs promising biomarkers for HF diagnosis, and their potential for HFpEF diagnosis has already been established [29]. To this end, we used miR-545, a biomarker for HFpEF, as a target to assess the detection capability of our DPNE system in serum samples.

**Figure 5:**
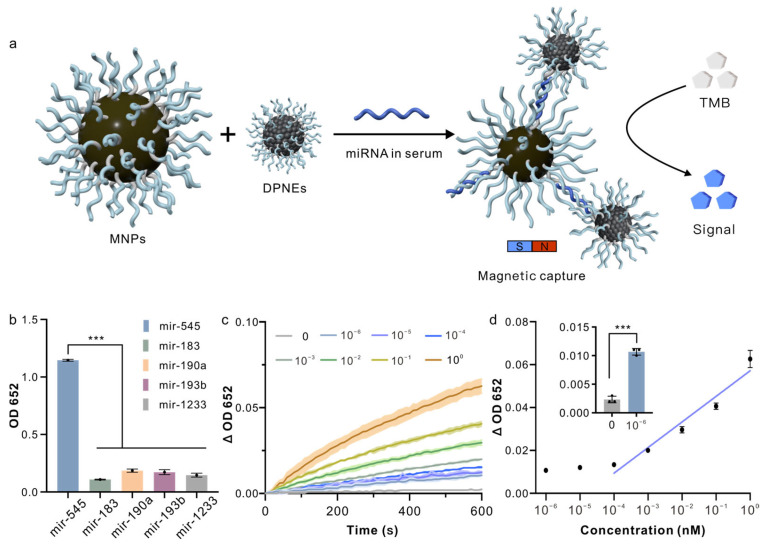
Direct detection of HFpEF-associated miRNA in serum using DPNEs. (**a**) Schematic of the DPNE detection process for miRNA in serum; (**b**) specificity validation, showing a prominent signal for only miR-545; (**c**) absorption kinetics for different concentrations of miR-545; (**d**) titration curve for miR-545 detection in serum by DPNEs, with absorbance signals from 10^−6^ nM miR-545 and blank serum (inset). Error bars, mean ± s.d. (*n* = 3). *** *p* < 0.001 by Student’s *t*-Tests.

We synthesized DPNEs with detection probes and MNPs conjugated with capture probes. In brief, we designed two diblock DNA strands composed of partially complementary sequences for miR-545 (light blue line) and a spacer sequence (polyT, gray line, sequence information in Table S1). These DNA strands were assembled onto the surfaces of PtNPs and MNPs via Au-S and biotin–avidin interactions, respectively. When miR-545 is present in the serum, the DPNEs bind to the MNPs and can be separated by a magnetic field. This separation results in an absorbance signal (OD 652) in the signal report solution. In the absence of miR-545, no DPNEs can be separated, yielding minimal signal output, thus enabling the detection of miR-545 in serum (Figure 5a).

We first evaluated the specificity of the DPNE system by comparing the signals generated from miR-183, miR-190a, miR-193b, miR-1233 and miR-545. The signals from other miRNAs were similar to the blank, while the signal from miR-545 was significantly elevated, approximately eight times stronger than the signals from other miRNAs (Figure 5b), confirming the specificity of the DPNE detection system. In addition to specificity, we also assessed the sensitivity of the DPNE system. Serum samples containing miR-545 at concentrations ranging from 10^−6^ to 1 nM were incubated with MNPs and DPNEs, with miR-545 negative serum samples used as negative controls. After magnetic separation, the particles were incubated with the signal report solution, and the absorbance changes were measured using a microplate reader. We defined the signal change as the difference between the absorbance at time t and time 0 (ΔOD 652 = OD 652 (T = t) − OD 652 (T = 0)). When miR-545 was absent, ΔOD 652 remained close to baseline, whereas as the concentration of miR-545 increased, ΔOD 652 increased progressively (Figure 5c). By correlating ΔOD 652 at T = 600 s with miR-545 concentration, we determined that the DPNEs system had a detection range from 10^-4^ to 1 nM, with a limit of detection as low as 10^−6^ nM (Figure 5d). Current clinical protocols predominantly rely on quantitative reverse transcription polymerase chain reaction (qRT-PCR) to evaluate serum miRNA levels for assessing HFpEF progression [30,31]. Recent advancements in miRNA detection, including digital PCR, CRISPR-based diagnostics, and next-generation sequencing (NGS), have aimed to enhance sensitivity, streamline workflows, reduce processing time, and minimize equipment dependency [32,33,34]. While these methods achieve remarkable sensitivity, they often require laborious sample pretreatment steps such as RNA extraction, library preparation or enzymatic amplification [35,36,37]. In contrast, our DPNE platform attains comparable sensitivity while entirely eliminating pretreatment requirements (Table S6). This advantage stems from two synergistic design features: (1) an anti-fouling DNA coating that selectively excludes interfering biomacromolecules and ionic contaminants through combined steric and electrostatic repulsion, and (2) magnetic separation-mediated target enrichment, which achieves miRNA recovery from raw biological matrices. Such technical advantages position the DPNEs platform as a promising solution for rapid screening and clinical diagnosis of HFpEF.

## 4. Conclusions

In summary, we have developed a DNA-engineered nanozyme coating that preserves the peroxidase-like catalytic activity of platinum nanoparticles in complex biological environments. The DPNEs enables ultrasensitive miRNA detection in serum with a detection limit of 1 fM. The protective mechanism operates through two synergistic effects: (1) electrostatic stabilization via the negatively charged DNA shell prevents salt-induced aggregation, and (2) molecular sieving through precisely controlled inter-oligonucleotide spacing selectively excludes macromolecular interferents while permitting substrate access to catalytic sites. This dual protection strategy maintains >95% of initial catalytic activity in serum compared to <30% retention for PtNPs.

The inherent programmability of DNA further enables the precise integration of molecular recognition elements through modular probe design. We demonstrated this capability through the specific detection of HFpEF-associated miR-545, achieving eight-fold signal discrimination against non-target miRNAs. The system’s clinical relevance is underscored by its ability to detect miRNA concentrations below established diagnostic thresholds (<0.1 pM) for cardiovascular pathologies.

This DNA-mediated surface engineering approach establishes a generalizable framework for developing robust nanozyme platforms, addressing three critical challenges in diagnostic applications: (1) colloidal stability under physiological ionic strength, (2) resistance to biofouling in protein-rich environments, and (3) programmable target recognition capability. The methodology could be readily extended to other catalytic nanomaterials and biomarker detection systems through rational DNA sequence design.

## Data Availability

Data are contained within the article and Supplementary Materials.

## Supplementary Materials

The following supporting information can be downloaded at https://www.mdpi.com/article/10.3390/bios15040205/s1, Figure S1: Catalytic kinetics of DPNEs with different DNA modification densities and different sequence length. Figure S2: Aggregation resistance of PtNPs and DPNEs at different salt ion concentrations; Figure S3: Catalytic activity of PtNPs and DPNEs at different salt ion concentrations; Figure S4: catalytic reaction kinetics of PdNPs and DPNEs (Pd). Table S1: Sequences of oligonucleotides used in this work; Table S2: Enzyme kinetic parameters of PtNPs in high-salt solution; Table S3: Enzyme kinetic parameters of DPNEs in high-salt solution; Table S4: Enzyme kinetic parameters of PtNPs and DPNEs in serum; Table S5: DLS measured particle size data. Table S6: Representative miRNA detection methods in recent years References [38,39,40] are cited in the Supplementary Materials.

## Abbreviations

The following abbreviations are used in this manuscript:

SNAs	Spherical nucleic acids
DPNEs	DNA-coated platinum nanozymes
PtNPs	Platinum nanoparticles
DLS	Dynamic light scattering
TMB	3,3′,5,5′-tetramethylbenzidine
BSA	Bovine serum albumin
HF	Heart failure
HFpEF	Heart failure with preserved ejection fraction
MNPs	Magnetic nanoparticles
