# Supplementary material for: DNA-Engineered Coating for Protecting the Catalytic Activity of Platinum Nanozymes in Biological Systems
Source: Biosensors (Basel). 2025 Mar 21;15(4):205. doi: 10.3390/bios15040205 (PMC12024773; doi:10.3390/bios15040205)
Supplement: Supplementary file 1 [file biosensors-15-00205-s001.zip › biosensors-3512340-supplementary.pdf]

biosensors

Supplementary Material

DNA-Engineered Coating for Protecting the Catalytic Activity of Platinum Nanozymes in Biological Systems

Lei Ren [1,2,3,†], Xia Liu [3,4,†], Shuai Tang [3], Yue Wang [1,2], Miao Yang [3], Linjie Guo [3], Jiang Li [3], Kai Jiao [3,*] and Lihua Wang [3,5,*]

[1] Division of Physical Biology, CAS Key Laboratory of Interfacial Physics and Technology, Shanghai Institute of Applied Physics, Chinese Academy of Sciences, Shanghai 201800, China； renlei@sinap.ac.cn (L.R.); wangyue0107@sinap.ac.cn (Y.W.)
[2] University of Chinese Academy of Sciences, Beijing 100049, China
[3] Institute of Materiobiology, College of Sciences, Shanghai University, Shanghai 200444, China; xliusinap@163.com (X.L.); stang@shu.edu.cn (S.T.); 17837702067@shu.edu.cn (M.Y.); guolinjie@shu.edu.cn (L.G.); lijiang80@shu.edu.cn (J.L.)
[4] Xiangfu Laboratory, Jiaxing 314102, China
[5] Shanghai Collaborative Innovation Center of Intelligent Sensing Chip Technology, Shanghai University, Shanghai 200444, China
* Correspondence: kjiao@shu.edu.cn (K.J.); wanglihua@shu.edu.cn (L.W.)
† These authors contributed equally to this work.

Table S1. Sequences of oligonucleotides used in this work.

Name	Sequence (5′-3′)
PolyT-6	TTTTTT-SH
PolyT-14	TTTTTTTTTTTTTT-SH
PolyT-18	TTTTTTTTTTTTTTTTTT-SH
PolyT-24	TTTTTTTTTTTTTTTTTTTTTTTT-SH
Capture probe	ACATTTACTGATTTTTTTTTT- Biotin
Detection probe	SH-TTTTTTTTTTCATCTAATAA
miR-545	UCAGUAAAUGUUUAUUAGAUGA
miR-1233	AGUGGGAGGCCAGGGCACGGCA
miR-190a	UGAUAUGUUUGAUAUAUUAGGU
mir-193b	AACUGGCCCUCAAAGUCCCGCU
miR-183	GUGAAUUACCGAAGGGCCAUAA

Table S2. Enzyme kinetic parameters of PtNPs in high-salt solution.

Name	V_{max}(nM/s)	K_m(mM)
10 mM Na^+/Mg^{2+}	489.435 ± 13.251	0.248 ± 0.026
100 mM Na^+	242.462 ± 19.528	0.565 ± 0.106
5mM Mg^{2+}	77.470 ± 8.192	0.975 ± 0.191

Table S3. Enzyme kinetic parameters of PtNPs in high-salt solution.

Name	V_{max}(nM/s)	K_m(mM)
0 mM Na+/Mg^{2+}	404.923 ± 12.958	0.108 ± 0.017
100 mM Na^+	423.382 ± 18.002	0.090 ± 0.021
250 mM Na^+	392.205 ± 3.175	0.124 ± 0.006
500 mM Na^+	388.103 ± 8.041	0.118 ± 0.012

1000 mM Na^+	392.615 ± 9.723	0.123 ± 0.015
5 mM $Mg2^+$	417.231 ± 3.692	0.107 ± 0.005
10 mM $Mg2^+$	403.740 ± 7.682	0.121 ± 0.011
20 mM $Mg2^+$	395.897 ± 5.887	0.107 ± 0.008
40 mM $Mg2^+$	400.001 ± 8.771	0.105 ± 0.012

Table S4. Enzyme kinetic parameters of PtNPs and DPNEs in BSA and serum.

Name	V_{max}(nM/s)	K_m(mM)
PtNPs (BSA)	489.435 ± 13.251	0.248 ± 0.026
PtNPs (serum)	245.333 ± 25.838	0.987 ± 0.192
DPNEs (BSA)	418.872 ± 7.590	0.093 ± 0.009
DPNEs (serum)	409.436 ± 6.523	0.0857 ± 0.008

Table S5. Hydrodynamic diameter of PtNPS and DPNEs measured by DLS.

Name	Average particle size (nm)
PtNPs	35.400 ± 0.174
PtNPs (5 mM Mg^{2+})	1102.333 ± 110.699
PtNPs (100 mM Na^+)	965.500 ± 58.711
PtNPs (BSA)	55.767 ± 0.689
PtNPs (serum)	71.457 ± 0.232
DPNEs	37.793 ± 0.191
DPNEs (5 mM Mg^{2+})	41.637 ± 0.162
DPNEs (100 mM Na^+)	43.027 ± 0.428
DPNEs (BSA)	37.917 ± 0.257
DPNEs (serum)	37.287 ± 0.416

Table S6. Representative miRNA detection methods in recent years.

Method	LOD	sample pretreatment	Detection duration（h）
$DISC_2$(5)[1]	5 fM	RNA extraction、miRNA extension、reverse transcription、PCR amplification	2-3
Digital PCR[2]	1 fM	RNA extraction、miRNA extension、reverse transcription、PCR amplification	3-4
SHERLOCK[3]	2 aM	RNA extraction、miRNA extension、reverse transcription、PCR amplification	3-4
SERS sensor[4]	0.15 pM	Cells cultured、RNA extraction	4-6
PEC[5]	83 fM	/	2-3
Fluorescence[6]	6 pM	/	2-3
Nanospheres[7]	70 pM	/	1-2
AgNCs[8]	38 pM	/	2-3
rGO/AuNPs[9]	1 pM	/	2-3

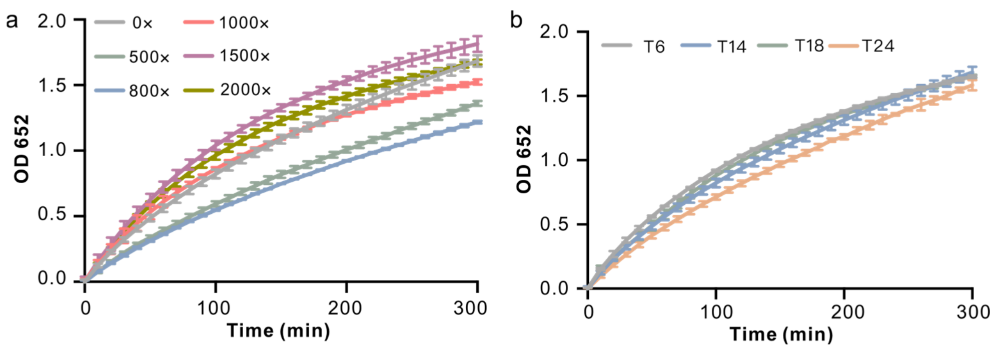

Figure S1. Catalytic kinetics of DPNEs with different DNA modification densities (a) and different sequence length (b).

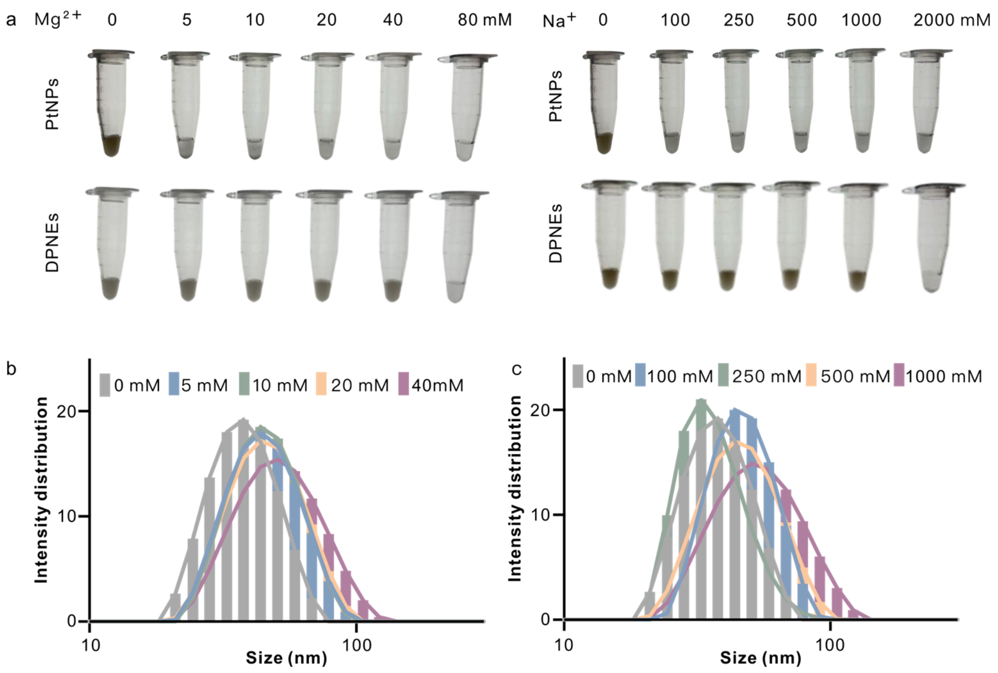

Figure S2. Aggregation resistance of PtNPs and DPNEs at different salt ion concentrations. (**a**) Bright-field images of PtNPs and DPNEs in solutions with varying salt ion concentrations. PtNPs aggregate in 5 mM Mg^{2+} and 100 mM Na^{+}, causing solution stratification and black aggregates at the bottom of the tube, while DPNEs remains as a homogeneous black solution in 40 mM Mg^{2+} and 1000 mM Na^{+}.; (**b**) Hydrodynamic radius distribution of DPNEs at different salt ion concentrations.

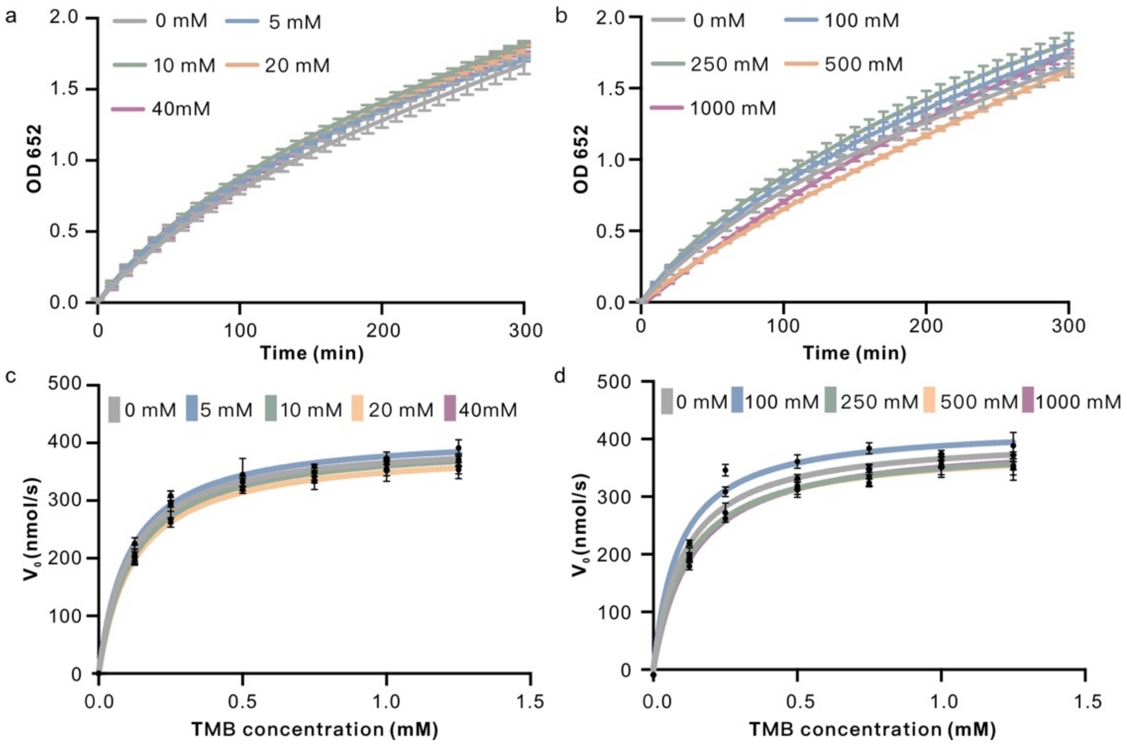

Figure S3. Catalytic activity of PtNPs and DPNEs at different salt ion concentrations. (**a**) Catalytic reaction kinetics of PtNPs; (**b**) Michaelis-Menten fitting curve for PtNPs; (**c**) Catalytic reaction kinetics of DPNEs; (**d**) Michaelis-Menten fitting curve for DPNEs.

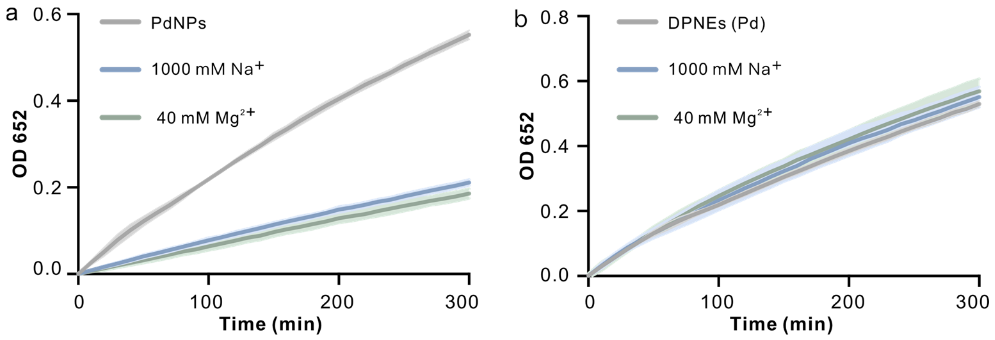

Figure S4. Catalytic reaction kinetics of PdNPs (a) and DPNEs (Pd) (b).

1. Dong, J.; Chen, G.; Wang, W.; Huang, X.; Peng, H.; Pu, Q.; Du, F.; Cui, X.; Deng, Y.; Tang, Z. Colorimetric PCR-Based microRNA Detection Method Based on Small Organic Dye and Single Enzyme. *Anal. Chem.* **2018**, *90*, 7107-7111, doi:10.1021/acs.analchem.8b01111.
2. Hindson, C.M.; Chevillet, J.R.; Briggs, H.A.; Gallichotte, E.N.; Ruf, I.K.; Hindson, B.J.; Vessella, R.L.; Tewari, M. Absolute quantification by droplet digital PCR versus analog real-time PCR. *Nat. Methods.* **2013**, *10*, 1003-1005, doi:10.1038/nmeth.2633.
3. Gootenberg, J.S.; Abudayyeh, O.O.; Kellner, M.J.; Joung, J.; Collins, J.J.; Zhang, F. Multiplexed and portable nucleic acid detection platform with Cas13, Cas12a, and Csm6. *Science* **2018**, *360*, 439-444, doi:10.1126/science.aaq0179.
4. Si, Y.; Xu, L.; Deng, T.; Zheng, J.; Li, J. Catalytic Hairpin Self-Assembly-Based SERS Sensor Array for the Simultaneous Measurement of Multiple Cancer-Associated miRNAs. *ACS Sensors* **2020**, *5*, 4009-4016, doi:10.1021/acssensors.0c01876.

5. Liu, S.; Cao, H.; Wang, X.; Tu, W.; Dai, Z. Green light excited ultrasensitive photoelectrochemical biosensing for microRNA at a low applied potential based on the dual role of Au NPs in TiO2 nanorods/Au NPs composites. *Nanoscale* **2018**, *10*, 16474-16478, doi:10.1039/C8NR05513K.
6. Liu, M.X.; Liang, S.; Tang, Y.; Tian, J.; Zhao, Y.; Zhao, S. Rapid and label-free fluorescence bioassay for microRNA based on exonuclease III-assisted cycle amplification. *RSC Advances* **2018**, *8*, 15967-15972, doi:10.1039/C8RA01605D.
7. Chen, T.; Xu, Y.; Wei, S.; Li, A.; Huang, L.; Liu, J. A signal amplification system constructed by bi-enzymes and bi-nanospheres for sensitive detection of norepinephrine and miRNA. *Biosens. Bioelectron.* **2019**, *124-125*, 224-232, doi:https://doi.org/10.1016/j.bios.2018.10.030.
8. Pan, M.; Liang, M.; Sun, J.; Liu, X.; Wang, F. Lighting Up Fluorescent Silver Clusters via Target-Catalyzed Hairpin Assembly for Amplified Biosensing. *Langmuir* **2018**, *34*, 14851-14857, doi:10.1021/acs.langmuir.8b01576.
9. Tian, R.; Ning, W.; Chen, M.; Zhang, C.; Li, Q.; Bai, J. High performance electrochemical biosensor based on 3D nitrogen-doped reduced graphene oxide electrode and tetrahedral DNA nanostructure. *Talanta* **2019**, *194*, 273-281, doi:https://doi.org/10.1016/j.talanta.2018.09.110.
